# A Systematic Review of Ecological Momentary Assessment Procedures of Self-Harm (With and Without Suicidal Intent) Studies in Adolescents and Young Adults

**DOI:** 10.3390/ijerph23010084

**Published:** 2026-01-07

**Authors:** Bethany Martin, Susan Rasmussen, Kirsten Russell, Megan Crawford, Spence Whittaker, Scott Thomson, Abbie Greenwood

**Affiliations:** Department of Psychological Sciences and Health, University of Strathclyde, 40 George Street, Glasgow G1 1QE, UK

**Keywords:** ecological momentary assessment, ambulatory assessment, experience sampling, systematic review, self-harm, suicide, non-suicidal self-injury, adolescence

## Abstract

Ecological momentary assessment (EMA) captures real-time data on thoughts, emotions, and behaviours within individuals’ natural environments. Although EMA has been increasingly used to examine self-harm, existing reviews have not focused specifically on adolescents. This systematic review examines EMA research on adolescent self-harm, focusing on methodological considerations and key risk and protective factors for self-harm. Five databases, plus pre-print, unpublished and grey literature sources, were searched up to 30 January 2024. Studies were included if published in English, used EMA methodology, included adolescents aged 10–24 years and measured suicidal ideation, suicidal behaviours, or self-harm. The review included 79 studies, published from 2009 to the present. Self-harm was associated with numerous risk factors, including negative affect, stress, interpersonal influences and sleep. EMA was generally well-accepted by adolescent participants, with high compliance rates. The findings highlight the value of EMA in capturing real-time fluctuations in self-harm and associated risk factors among adolescents. EMA demonstrates strong potential for improving understanding and prediction of self-harm; yet challenges remain, including variability in study designs and a lack of clear reporting of the methodologies. Future research should focus on standardising methodologies, increasing participant diversity, and exploring the clinical utility of EMA in early intervention and prevention strategies.

## 1. Introduction

Suicide is the second most common cause of death globally among individuals aged 15 to 29 years [1] and one of the strongest predictors of death by suicide is a history of self-harm. For the purposes of this review, self-harm is defined as “intentional self-poisoning or injury, irrespective of the apparent purpose of the act” [2]. Therefore, this review encompasses self-harm thoughts and behaviours, non-suicidal self-injury (NSSI), self-poisoning, suicide attempts, suicidal ideation and suicide plans. Given the complexity and overlap in terminology across the literature, this review adheres to the specific terminology used by authors when describing thoughts and behaviour within each study, to ensure clarity and fidelity to the original operationalisation. Self-harm is a prevalent issue among adolescents [3], with self-harm rates rising over the past decades despite the ongoing research and prevention efforts [4,5,6]. Self-harm thoughts and behaviour among adolescents represent a concerning and complex issue with significant implications for mental health and well-being.

Understanding the aetiology of self-harm, as well as the proximal risk and protective factors, is important to identify effective prevention targets. The risk of self-harm is highly dynamic, fluctuating over short periods of time; yet much of our understanding of self-harm thoughts and behaviours is derived from traditional cross-sectional and longitudinal studies [7,8]. These approaches fail to capture the variable and time-sensitive nature of self-harm, limiting our ability to accurately predict, intervene and prevent self-harm in a timely manner. Franklin et al. [9] meta-analysis highlighted that our ability to predict suicidal behaviour has not significantly improved. This reliance on traditional research methods may contribute to the current gaps in understanding any short-term mechanisms that perpetuate, exacerbate or stop self-harm. Consequently, advancing beyond retrospective methods to capture between- and within-person differences in a time-sensitive manner is important.

Ecological momentary assessment (EMA) offers a promising methodological approach by capturing real-time data on thoughts, emotions, behaviours, experiences, and diverse other factors in participants’ natural environments [10]. This approach accounts for variability in factors by assessing participants repeatedly, typically using digital self-report diaries, throughout the day. The repeated and flexible nature of EMA over short periods is particularly valuable in mental health contexts, as it allows for the monitoring of the fluctuations in self-harm risk. Research utilising EMA not only captures risk factors but also allows for the exploration of protective factors and adaptive coping strategies utilised by adolescents to mitigate self-harm tendencies. Further, EMA methods seek to reduce recall bias and increase ecological validity over retrospective study designs [10,11]. The potential of collecting data via repeated measurements on mobile devices to investigate self-harm has started to gain widespread recognition, as evidenced by the growing body of research utilising EMA and similar methodologies to study these behaviours [11,12,13].

EMA encompasses a collection of methods that share a repeated assessment approach to capturing individuals’ behaviours, thoughts, and emotions that is highly adaptable depending on the study’s objectives. Assessments in EMA can be scheduled in different ways: event-contingent assessments are triggered by specific events, such as when a participant is about to engage in or has just engaged in a behaviour, while time-based assessments are scheduled at fixed or random intervals throughout the day. Daily diaries represent a type of EMA in which participants complete assessments once per day, usually at the end of the day. EMA methods initially relied on pen-and-paper diaries, requiring participants to carry assessment booklets and return these to the research team after data collection. With technological advancements, EMA studies transitioned to personal digital assistants (PDAs), and more recently, the widespread availability of smartphones has significantly enhanced the feasibility and accessibility of this methodology [14]. This enables the verification of entry times, allowing more detailed consideration of compliance and provides the opportunity for real-time safety monitoring in high-risk samples. Furthermore, considering the digital habits of adolescents, a population at risk for self-harm, EMA emerges as a potentially appropriate and effective methodology for studying this group.

The acceptability of EMA methodology within adolescent samples remains uncertain. While there are recent systematic reviews in this area, these encompass all age groups [11,12,13,15]. This rapidly advancing field also requires ongoing updates, making a review focused on adolescent self-harm EMA studies timely and necessary to synthesise the expanding base of literature. This current review aims to address the gap by specifically focusing on how EMA has advanced our understanding of self-harm risk among adolescents in their daily lives. By synthesising these findings, this review aims to guide the design of future EMA studies tailored to adolescents, ultimately enhancing the understanding of adolescent self-harm thoughts and behaviours.

Therefore, this review aims to (a) examine the methodological approaches and procedures used in EMA studies to advance understanding of adolescents’ self-harm risk within the context of daily life and (b) synthesise the results of these investigations.

## 2. Methods

This systematic review was conducted in accordance with the Preferred Reporting Items for Systematic Reviews and Meta-Analyses (PRISMA) guidelines; the completed PRISMA 2020 Checklist is provided in the Supplementary Materials. The protocol for this review was pre-registered on PROSPERO (CRD42024487468). The only deviation from the registered protocol was the inability to access EThOS, the British Library’s online database of doctoral theses, at the time of record searching due to a cyber attack.

### 2.1. Search Strategy

A search strategy was employed to identify studies using EMA in adolescent self-harm research. The search was conducted across Web of Science Core Collection, Embase, PsycINFO, CINAHL, and PubMed. To ensure a thorough search, pre-print repositories PsyArXiv and medRxiv were also included, alongside grey literature sources from CORE. Unpublished manuscripts and ongoing projects were identified via NIH RePORTER and the Open Science Framework (OSF) registration site. Where grey literature overlapped with subsequently published peer-reviewed articles, duplicates were manually identified and the published version was retained. For any identified conference abstracts and records of ongoing projects, corresponding authors were contacted by email to request additional information. Additional relevant articles were searched for by manually reviewing the reference lists of all included studies. The search included studies published or registered up until the date of the search, 30 January 2024, without restrictions on the publication period.

The search terms were formulated based on the concepts of EMA, self-harm, and the adolescent population. Terms included variations and synonyms of “Ecological Momentary Assessment,” “self-harm,” and “adolescents,” adapted from the search strategy used by Gee et al. [12]. The complete search strategy is provided in Appendix A.

### 2.2. Eligibility Criteria

Eligibility criteria were guided using the SPIDER framework (sample, phenomenon of interest, design, evaluation, research type) [16]. Inclusion criteria were as follows: (1) published in English, (2) studies using ecological momentary assessment methodology, (3) research conducted with samples of adolescents ranging from 10 to 24 years old or with a mean age falling between 10 and 24 years, (4) EMA studies that included measures on suicidal ideation, suicidal behaviours or self-harm.

Studies were excluded if they were published in languages other than English, commentaries, editorials, qualitative studies or studies that provide insufficient information on study procedure or EMA methodology.

### 2.3. Study Selection

**Figure 1:**
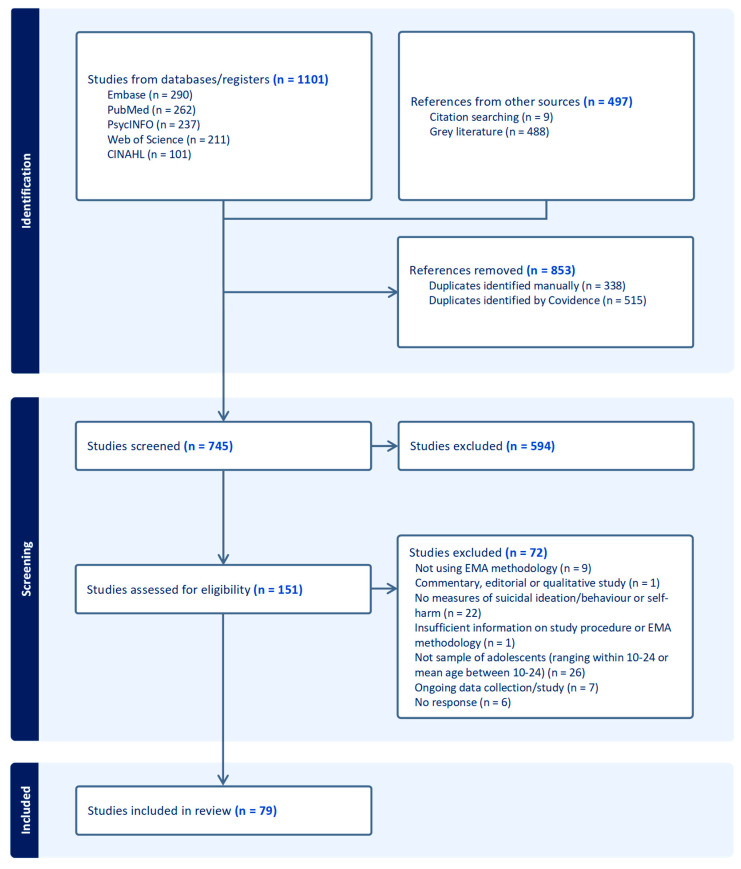
Flowchart of Systematic Review.

All titles and abstracts identified through the search strategy were independently screened by two reviewers to remove irrelevant records. Full-text articles were reviewed for inclusion by two reviewers, deeming 79 eligible. The eleven discrepancies between reviewers regarding study eligibility were resolved through discussion within the research team. The selection process is documented using a PRISMA flow diagram, see Figure 1 below.

### 2.4. Data Extraction

Data extraction was performed by four reviewers using a standardised data extraction form, ensuring each record was reviewed by two separate individuals and consensus confirmed. Any disagreements in data extraction were resolved by discussion or by involving a third reviewer. The extracted data included the following:

Study Information: Title, author(s), year of publication, journal, country, and type of publication.

Sample Characteristics: Total number of participants, mean age, age range, gender distribution, educational background, socioeconomic status, ethnicity, presence of psychiatric disorders, and details on self-harm and suicidal thoughts or behaviours.

EMA Methodology: Details of the EMA protocol, including participant training, prompt strategy, frequency and timing of prompts, duration of assessments, and technological platforms used.

Outcomes: Associations between self-harm and other variables, frequency of self-harm episodes, methods used, and alternative coping mechanisms.

Response and Compliance: Information on recruitment strategies, participant retention, response rates, and compliance with EMA protocols.

Due to the diverse nature of populations and heterogeneity of EMA methodology, meta-analytic synthesis was considered unsuitable for this review. The evidence was synthesised narratively.

### 2.5. Quality Assessment Methodology

The quality of the included studies was assessed using a composite reporting quality assessment tool for experience sampling method (ESM) studies [17]. This tool is available on OSF: https://osf.io/dxvsh/ (accessed on 8 July 2024). Each study was rated by one reviewer, and uncertainties were resolved by consulting a second reviewer.

## 3. Results

### 3.1. Search Results and Study Characteristics

The search retrieved a total of 1598 records, of which 853 were duplicates. Of the 745 unique records, 594 were excluded after screening titles and abstracts, and a further 72 were excluded after assessing the full text for eligibility. This resulted in 79 papers included in this review.

#### 3.1.1. Publication and Location Trends

**Figure 2:**
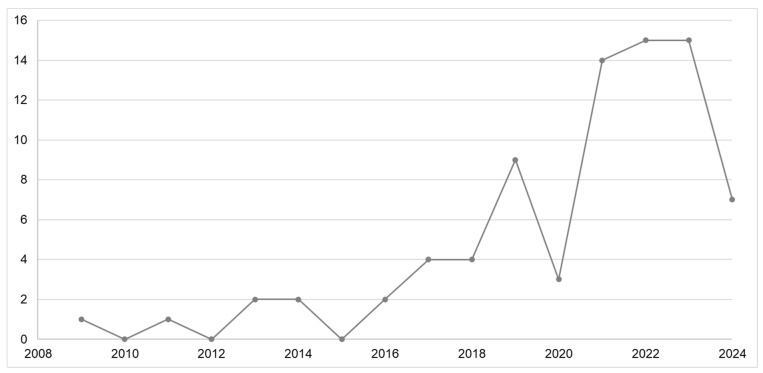
Number of Publications over Time.

There has been an increase in EMA publications, with a notable growth after 2018, as shown in Figure 2. In this review, 12 records (15.2%) were identified between 2009 and 2017. From 2018 onwards, 67 records (84.8%) were found. Most studies were conducted in the United States (*n* = 54; 68.4%). Other countries included the United Kingdom (*n* = 3; 3.8%), Canada (*n* = 3; 3.8%), Belgium (*n* = 3; 3.8%), Germany (*n* = 3; 3.8%), Australia (*n* = 2; 2.5%), Spain (*n* = 2; 2.5%), the Netherlands (*n* = 1; 1.3%), Israel (*n* = 1; 1.3%) and Korea (*n* = 1; 1.3%). For six studies (7.6%), the location was not clear.

#### 3.1.2. Sample Characteristics

Recruitment settings included community (*n* = 12; 15.2%), clinical (*n* = 28; 35.4%), university (*n* = 15; 19.0%), and combined (*n* = 22; 27.8%) settings. While most studies were conducted in natural settings, two studies (2.5%) involved participants completing EMA assessments in clinical in-patient environments.

Sample sizes varied from 10 to 1210 participants, with an overall mean age of 18.79 years (ranging from an average of 12.03 to 23.92). The participants in this research were predominantly White (66.9%) and female (76.6%). Compliance ranged broadly across studies from 38% to 92%.

#### 3.1.3. EMA Schedules and Tools

The average number of days of observation was 22.38 (ranging from 2 days to 6 months), with an average of 3.5 prompts each day. A number of records in this review (*n* = 30; 38.0%) were daily diary designs, while the maximum number of prompts per day was 12. The majority used a signal-contingent prompt strategy (*n* = 46; 58.2%), followed by mixed or combined strategies (*n* = 22; 27.8%), interval (*n* = 6; 7.6%), and one study (1.3%) used an event-contingent only strategy. Mobile phones were the primary data collection tool (*n* = 60; 76.0%), while five studies (6.3%) used PDA devices, and three studies (3.8%) collected pen and paper diaries. A further four studies (5.1%) utilised either emailed links or online platforms for the assessments, and ten studies (12.7%) did not specify the data collection tool used. Seven records (8.9%) included actigraphy or sensor wristbands, alongside the EMA assessments.

#### 3.1.4. Measurement of Self-Harm

Self-harm was operationalised across studies using a variety of self-report items, reflecting heterogeneity in the conceptualisation of this construct. Across the 79 studies reviewed, the EMA self-harm measures varied significantly. Table 1 provides an overview of self-harm measures used in each study. The terminology used for constructs (e.g., suicidal ideation, urge or intent) reflects the original source’s wording. Where available, the number of items and/or the source of each measure are reported. Non-suicidal self-injury (NSSI) was the most frequently assessed, appearing in 46 (58.2%) of the studies. Suicidal ideation was measured in 35 studies (44.3%), with a further 6 measuring suicide urge (7.6%). Suicidal behaviours (e.g., suicide attempts) were assessed in 10 studies (12.7%). Self-harm, as defined by NICE [2], was only captured in a small minority of the studies (*n* = 5; 6.3%). A number of studies, however, measured both NSSI and suicidal ideation, e.g., [18,19,20,21]. This highlights a predominant focus on NSSI and suicide within the current EMA research.

Studies used single-item or brief multi-item measures to capture relevant thoughts and/or behaviours. NSSI behaviour was often measured with an item asking whether the participant had engaged in self-injury since the last prompt, e.g., [22]. Further, some studies included separate items to capture both urges and engagement in NSSI [23,24]. A subset of studies also included assessment of NSSI method and/or function, asking participants to indicate reasons for self-injurious behaviour when it occurred [25,26]. Presence of suicidal ideation was similarly assessed via brief scales, sometimes including Likert formats to consider thought intensity or duration [27,28]. Few studies distinguished between passive and active ideation, e.g., [29]. Despite the diversity in measurement, a common trend was the brevity of items to minimise participant burden in these real-time contexts.

### 3.2. Quality Assessment Results

Table 2 provides a summary of the reporting quality across the EMA studies. The majority of studies justified using this methodological approach to some extent (*n* = 67; 84.8%), but the reporting of design details was frequently limited. In particular, only a small number of studies provided any rationale for the choice of sampling design (*n* = 6; 7.6%) or sampling density (*n* = 23; 29.1%). Further, the full text of items included in EMA assessments was often not provided and the reliability and validity of the items were infrequently thoroughly addressed. Most studies did not fully comply with the reporting of results; reflections on factors such as prompt delivery (*n* = 5; 6.3%), latency (*n* = 3; 3.8%), and the influence of demographic variables on missing data (*n* = 17; 21.5%) were uncommon. Lastly, while data (*n* = 19; 24.0%) and materials (*n* = 12; 15.2%) were sometimes available upon request, few studies proactively made them publicly available.

**Table 1:** Characteristics and Main Findings of EMA studies.

Lead Author and Publication Date	Sample Size and Description	Mean Age (Range)	Percentage Female	EMA Methods (Duration, Prompt Strategy, Prompt Frequency, Tool)	Compliance	EMA Self-Harm Measure (Number of Items, Original Source)	Main Findings
Al-Dajani 2022 [27]	78 adolescents after psychiatric hospitalisation	15.19 (13–17)	67.9%	4 weeks of signal (1/day) sampling with mobile phone	72.4%	Suicidal Urge Intensity (1 item [30])	The interaction between burdensomeness and thwarted belongingness was significantly associated with increased same-day suicidal ideation but not next-day ideation. The only significant predictor of next-day ideation was higher burdensomeness reported the previous day.
Al-Dajani 2022 [31]	78 adolescents after psychiatric hospitalisation	15.19 (13–17)	69%	4 weeks of signal (1/day) sampling with mobile phone	72.4%	Suicidal Ideation Urges (2 items based on [32])	Seeking professional support and perception of coping helpfulness were associated with lower next-day suicidal urges, even after accounting for previous-day urges. Perceived coping helpfulness was particularly effective in reducing daily suicidal urge intensity at both within- and between-person levels. Support-seeking coping strategies were generally linked to less severe suicidal thoughts; however, professional support seeking was the least endorsed coping strategy.
Aleva 2023 [33]	Young people with past-year self-harm			28 days of signal (5/day) sampling with mobile phone		Self-harm Thoughts and Behaviours (1 item)	N/A (protocol).
Andrewes 2017 [34]	107 young people with BPD	18.1 (15–25)	83.2%	6 days of signal (6/day) sampling with mobile phone	51.6%	Self-injurious Thoughts (1 item) and NSSI Behaviour (1 item)	The number of negative complex emotions experienced before and after self-injurious thoughts and behaviour rose and fell alongside self-reported distress levels. However, changes in conflicting emotions did not align with distress levels and did not significantly increase before or decrease after self-injurious thoughts or NSSI. The intensity of distress reported while engaging in self-injurious thoughts and that reported during NSSI were not significantly different. Participants less accepting of their negative emotions were more likely to experience an increase in negative complex emotions before engaging in self-injurious thoughts and NSSI.
Andrewes 2017 [35]	107 young people with BPD	18.1 (15–25)	83.2%	6 days of signal (6/day) sampling with mobile phone	51.6%	NSSI Thoughts and Behaviour (5 items)	The most commonly reported motive for engaging in NSSI was affect regulation. Changes in negative affect significantly increased prior to NSSI and reduced following NSSI, whereas positive affect significantly reduced prior to NSSI and increased following NSSI. The duration between self-injurious thoughts, changes in affect, and engagement in NSSI was highly variable.
Armey 2011 [36]	36 university students with self-reported history of NSSI	18.7 (18–35)	75%	7 days of signal (6/day) and event (self-initiate after experience of any NSSI) sampling with PDA device	38%	NSSI Behaviour (3 items)	Individuals who endorsed NSSI behaviour experienced increases in negative affect prior to the behaviour, which peaked during the episode and faded gradually in the hours following the episode. These changes in affect were detected only at times in which individuals engaged in NSSI and were absent for individuals with NSSI history that did not report NSSI behaviour during the study. Further, these changes in negative affect were, on average, detectable hours prior to the NSSI event.
Bentley 2017 [37]	10 adults with NSSI disorder	21.3 (18–30)	90%	4 weeks of signal (1/day) and event (self-initiated after NSSI urge or act) sampling with mobile phone		NSSI Urge and Behaviour	N/A (no EMA-related findings reported; results focused on intervention).
Bentley 2024 [38]	236 adolescents after psychiatric hospitalisation	14.87 (12–18)	64.4%	6 months of interval and signal (6/day for 3 months, then 1/day for 3 months) sampling with mobile phone		Suicidal Intent (1 item) and Suicidal Urge (1 item)	EMA completion did not differ after response-contingent interventions. Adolescents had a significantly increased likelihood, relative to the adult sample, to lower initially high-risk ratings to below the high-risk threshold after automated pop-up messages.
Berghoff 2022 [39]	103 young people with and without NSSI history	20.35 (18–35)	77.7%	14 days of interval (1/day) sampling	82.8%	NSSI Behaviour	Young adults with recent, recurrent NSSI reported more negative emotions following daily stressors. Overall, the groups with and without NSSI history did not differ in the frequency of reporting positive or negative interpersonal experiences.
Bresin 2013 [40]	67 university students with past-year NSSI	19.58	56.7%	14 days of signal (1/day) sampling through email	65.4%	NSSI Urge (1 item based on [32])	Negative urgency moderated the association between daily sadness, but not guilt or general negative affect, and urge to engage in NSSI.
Briones-Buixassa 2021 [41]	64 young people with BPD and young people without BPD	BPD = 23.62 (18–33) STD = 19.84 (18–33) HC = 22.65 (18–33)	BPD = 90% STD = 84.2% HC = 86.4%	15 days of signal (3/day) sampling and event (self-initiated after engaging in self-injury) with mobile phone	BPD = 73.7% STD = 60.5% HC = 99.9%	NSSI (4 items)	Momentary frustration directly predicted NSSI behaviour. Momentary guilt and anger only predicted NSSI when interacting with more stable traits of borderline pathology and negative emotional symptoms. Higher decentering capacity reduced the risk of self-injury and weakened the association between momentary sadness and NSSI.
Burke 2021 [42]	64 university students with repetitive NSSI history	20	92.2%	10 days of signal (4/day) sampling with mobile phone	88%	NSSI Urge (1 item) and NSSI Behaviour (1 item)	Trait and aggregated state self-punishment predicted NSSI urges. Momentary self-criticism and self-punishment were prospectively associated with NSSI urge intensity.
Burke 2021 [43]	60 university students with repetitive NSSI history	20.13 (18–26)	91.7%	10 days of signal (4/day) sampling with mobile phone	88.9%	NSSI Urge (1 item) and NSSI Behaviour (1 item)	Emotional response inhibition to self-harm images interacted with momentary negative affect to predict the strength of NSSI urges, after adjusting for emotional response inhibition to neutral images. Negative affect and urgency were associated with NSSI urge.
Burke 2022 [44]	64 university students with NSSI history	20.05 (18–26)	92.2%	10 days of signal (4/day) sampling with mobile phone	87.4%	NSSI Urge (1 item)	Behavioural approach system facets (drive and reward responsiveness) were associated with NSSI urge severity, but were not predictive of NSSI urge severity.
Burke 2022 [45]	119 university students with and without NSSI history	19.87 (18–26)	89.1%	10 days of signal (4/day) sampling with mobile phone	1132 of 1190 days of data completed	Momentary NSSI Urge Severity (1 item)	Individuals with a repetitive NSSI history were more likely to experience sleep irregularity than those without a history of NSSI. Higher sleep irregularity predicted higher daily NSSI urge severity, even after controlling for sleep duration, sleep timing, and negative affect.
Christensen 2023 [23]	93 young people with past-month NSSI	23.52 (18–34)	55.9%	2 weeks of signal (6/day) sampling	75.5%	NSSI Urge (1 item) and NSSI Behaviour (1 item)	Baseline- and EMA-reported perceived emotional social support were positively correlated. EMA-reported social support was related to EMA-reported NSSI urges but not NSSI behaviours.
Clark 2024 [28]	31 LGBTQ+ young people with past-year suicidal thinking	Median 21 (IQR = 18–22)	71%	28 days of signal (3/day) sampling with mobile phone	Median 90.5%	Suicidal Ideation Intensity (3 items based on [46])	Real-time exposure to LGBTQ+ negative news or media, but not general negative news or media, was significantly associated with increased intensity of active suicidal ideation, passive suicidal ideation, and thoughts of self-harm.
Clark 2025 [47]	50 LGBTQ+ young people with past-year suicidal thinking	18.38 (13–24)	76%	28 days of signal (3/day) sampling with mobile phone	80.2%	Active Suicidal Ideation Intensity (1 item based on [48,49]), Passive Suicidal Ideation Intensity (1 item based on [48,49]) and Non-Suicidal Self-Injury Ideation Intensity (1 item based on [42,50])	The EMA protocol was shown to be feasible, with participants showing high compliance with the EMA surveys. Weekly feedback surveys reflected generally high acceptability, as participants reported that the EMA surveys were easy to complete, private, understandable, and minimally burdensome.
Coppersmith 2019 [51]	53 adults with past-year suicide attempt	23.52 (18–39)	77.1%	28 days of signal (1/day) sampling with mobile phone	44% completed at least 21 days	Suicidal Ideation (3 items based on [52])	Social support was associated with same-day suicidal ideation and next-day suicidal ideation, even after adjusting for the effects of sadness, burdensomeness, and thwarted belonging. However, social support was not significantly associated with next-day suicidal ideation when adjusting for same-day suicidal ideation.
Coppersmith 2022 [53]	22 participants (7 adult psychiatric outpatients with NSSI disorder in sample 1 and 15 community-based adolescents in sample 2)	Sample 1 = 22 Sample 2 = 17 (12–19)	Sample 1 = 86% Sample 2 = 93%	Sample 1 = average 86 days of interval (1/day) and event (self-initiate whenever experiencing an urge to engage in or engaged in NSSI) sampling with mobile phone (varied on treatment-length) Sample 2 = 14 days of interval (2/day) and event (self-initiate when experienced a self-injurious thought or behaviour) sampling with PDA	Sample 1 = 94.1% of days	Sample 1 = NSSI (3 items) Sample 2 = NSSI (2 items)	Most participants exhibited changes in NSSI functions over time, reflecting high within-person variability. While a single function was typically reported per NSSI episode, some participants endorsed multiple functions co-occurring for the same episode. The most frequently endorsed function, and the only function reported by all participants, was automatic negative reinforcement.
Czyz 2018 [30]	34 adolescents after psychiatric hospitalisation	15.5 (13–17)	76.5%	28 days of signal (1/day) sampling with mobile phone	69.4%	Suicidal Ideation and Behaviour (7 items based on [54])	Adolescents without a history of suicide attempts were half as likely to adhere. Diaries were acceptable and most adolescents reported no change or a positive change in their thoughts and mood after daily surveys.
Czyz 2019 [55]	36 adolescents after psychiatric hospitalisation	15.42 (13–17)	78.8%	28 days of signal (1/day) sampling with mobile phone	69%	Suicidal Ideation (3 items based on [54])	N/A (no EMA-related findings reported; results focused on intervention).
Czyz 2019 [56]	34 adolescents after psychiatric hospitalisation	15.5 (13–17)	76.5%	4 weeks of signal (1/day) sampling with mobile phone	69.4%	Suicidal Ideation (4 items based on [54])	Same-day suicidal ideation frequency and duration were associated with connectedness, burdensomeness and hopelessness. The interaction between connectedness with both burdensomeness or hopelessness was significantly associated with next-day suicidal ideation frequency and duration.
Czyz 2019 [18]	34 adolescents after psychiatric hospitalisation	15.5 (13–17)	76.5%	4 weeks of signal (1/day) sampling with mobile phone	69.4%	Suicidal Ideation (3 items based on [54]) and NSSI Behaviour (2 items)	NSSI was positively associated with suicidal ideation at the between- and within-person level. When NSSI and suicidal thoughts co-occurred, adolescents used NSSI to cope with thoughts of suicide in nearly all instances. Adolescents who utilised more coping strategies in general had lower probability of NSSI. The likelihood of NSSI also decreased when adolescents perceived coping to be helpful.
Czyz 2021 [57]	32 adolescents after psychiatric hospitalisation	15.4 (13–17)	75%	28 days of signal (1/day) sampling with mobile phone	76.3%	Suicidal Ideation (1 item), Suicidal Ideation Duration (1 item based on [54]) and Self-efficacy to refrain from Suicidal Action (1 item from [58])	Models derived from combinations of risk factors produced higher predictive accuracy for short-term suicide risk than single risk factor models.
Czyz 2021 [59]	78 adolescents after psychiatric hospitalisation	15.19 (13–17)	67.9%	4 weeks of interval (1/day) sampling with mobile phone	74.2%	Suicidal Ideation (3 items based on [32,54]) and NSSI Behaviour and Functions (7 items based on [60,61])	The likelihood of NSSI was significantly higher for adolescents who had more enduring and severe suicidal ideation, relative to those with lower levels of ideation. Further, when adolescents experienced more enduring and intense suicidal ideation, relative to their own typical levels, the probability of NSSI also increased. Suicide thoughts co-occurred with NSSI endorsements the majority of time.
Czyz 2022 [62]	139 patients (61 psychiatric inpatients in sample 1 and 78 adolescents after psychiatric hospitalisation in sample 2)	Sample 1 = 15.16 (13–17) Sample 2 = 15.19 (13–17)	Sample 1 = 68.9% Sample 2 = 67.9%	Sample 1 = Max. 10 days of signal (5/day) sampling with mobile phone in clinical setting (varied on duration of hospitalisation) Sample 2 = 4 weeks of signal (1/day) sampling with mobile phone	Sample 1 = 65.8% Sample 2 = 74.2%	Suicidal Ideation (2 items based on [32,54])	Suicidal thoughts followed three distinct longitudinal trajectories, characterised by low, declining, or persistently high ideation in terms of frequency and urge severity. The persistent ideation trajectory showed consistently increased within-person variability. The persistent ideation group was differentiated by higher hopelessness and lower coping self-efficacy compared to the declining ideation group, and by an overall more severe clinical presentation relative to the low ideation group.
Czyz 2023 [63]	78 adolescents after psychiatric hospitalisation	15.19 (13–17)	67.9%	4 weeks of signal (1/day) sampling with mobile phone	74.2%	Suicidal Thoughts (1 item based on [54]) and Suicidal Ideation Duration (1 item based on [54])	In working towards developing a decision tool from machine learning to predict next-day suicidal ideation, the best-performing model included ideation duration, hopelessness, burdensomeness, and self-efficacy to refrain from suicidal action.
Czyz 2023 [64]	102 young people with recent emergency department visit for suicide risk	20.9 (18–25)	66.6%	8 weeks of signal (4/day) sampling with mobile phone	64.4%	Suicidal Ideation (3 items based on [54]), Death Thoughts (2 items), Self-efficacy to refrain from Suicidal Action (1 item from [58]) and NSSI (1 item)	EMA data produced a better-performing model and showed good accuracy for short-term prediction of suicidal ideation, compared to sensor data models. Passive sensing features did not improve prediction when combined with EMAs. Suicidal ideation-related features were the strongest predictors of next-day ideation.
Defayette 2023 [65]	42 university students with self-harm thoughts	19.6 (18–23)	73.8%	28 days of interval (1/day) and signal (5/day) sampling with mobile phone	71.8%	Suicidal Ideation Presence and Intensity (2 items based on [32,46]) and Suicidal Behaviour (2 items based on [32,46])	Feelings of exclusion were associated with increases in same-day suicidal ideation intensity. Increases in negative peer events were associated with increased odds of subsequent suicidal ideation among individuals with very low IL-6 activity. IL-6 activity moderated the relationship between number of negative peer events and subsequent, but not concurrent, presence of suicidal thoughts.
Esposito 2022 [19]	41 adolescents who had recently received acute psychiatric care for suicide risk	14.9 (12–17)	61%	28 days of interval (1/day), signal (3 to 6/day) and event (self-initiate if self-harm thoughts or behaviours occurred between assessments) sampling with mobile phone	62%	NSSI (1 item), and Suicidal Ideation and Suicide-Related Behaviours (4 items)	There were no statistically significant differences in self-injurious thoughts and behaviour frequency by report method. Adolescents reported significantly more frequent suicidal ideation during the interview, compared to more severe suicidal ideation reported via EMA surveys. Reasons for not reporting thoughts or behaviours in EMA were avoiding additional EMA questions and uncertainty about consequences from EMA reporting.
Fischer 2022 [66]	37 young people with PTSD diagnosis	18.2 (14–21)	89%	Variable duration based on treatment length of diary card (1/day) sampling in clinical setting	85.7%	Suicidal Ideation (1 item) and Self-injury (2 items)	N/A (no EMA-related findings reported; results focused on intervention).
Fitzpatrick 2020 [24]	47 actively self-injuring young people	19.1 (15–21)	68.1%	2 weeks of signal (5/day) sampling and event (self-initiated after engaging in self-injury) with mobile phone	85.1% of participants completed at least 80% of signal-contingent entries	NSSI Thoughts and Behaviour (8 items based on [32])	Higher NSSI thought intensity increased the likelihood of self-injury. Thought intensity and duration interacted to predict NSSI frequency, duration, number of methods, and likelihood of cutting. During NSSI episodes, the presence of NSSI thoughts was linked to higher frequency of behaviour.
Franssens 2023 [67]	131 young people	20.9	64.9%	14 days of signal (1/day) sampling	66%	NSSI Thoughts (1 item)	Daily negative emotionality, disinhibition, interpersonal distrust, and rejection sensitivity were positively associated with daily NSSI thoughts. Those who generally experienced higher negative emotionality, interpersonal distrust, or rejection sensitivity were at greater overall risk of self-harming thoughts.
Franz 2021 [68]	46 adults with past-year suicide attempt	23.4 (18–38)	78.2%	28 days of signal (1/day) sampling with mobile phone		Suicidal Thinking (3 items based on [52])	At higher levels of reappraisal, there was a weaker association between stress and suicidal thoughts. However, at higher levels of expressive suppression, there was also a weaker association between stress and suicidal thoughts.
Gerner 2023 [69]	43 university students with suicidal thoughts	19.12	69.8%	10 days of signal (5/day) sampling with mobile phone	85.8%	Suicidal Desire (1 item [46])	Hopelessness about one’s thwarted belongingness and interpersonal hopelessness about perceived burdensomeness on others showed greater consistency over time compared to general hopelessness. Hopelessness about one’s thwarted belongingness, interpersonal hopelessness about perceived burdensomeness on others and the interaction between these constructs were significant predictors of same-day and next-day suicidal desire. Hopelessness about one’s thwarted belongingness and interpersonal hopelessness about perceived burdensomeness on others were not stronger unique predictors of suicidal desire than general hopelessness.
Glenn 2021 [70]	48 adolescents that had recently received acute psychiatric care for suicide risk	14.96	64.6%	28 days of interval (1/day) and signal (3 to 6/day) sampling with mobile phone		Suicidal Thoughts (4 items based on [32,46])	The assessed indices of sleep problems via sleep diary (greater sleep onset latency, nightmares, ruminative thoughts before sleep) predicted next-day suicidal thinking, after controlling for baseline depression and daily sadness. Sleep indices assessed objectively (via actigraphy) were either not related to suicidal thinking or were related in the opposite direction than expected.
Glenn 2022 [71]	48 adolescents that had recently received acute psychiatric care for suicide risk	14.96	64.4%	28 days of interval (1/day) and signal (3 to 6/day) sampling with mobile phone		Suicidal Thoughts (4 items based on [32,46])	There were significant direct effects between family thwarted belongingness and suicidal thoughts, as well as friend thwarted belongingness and suicidal thoughts. Family thwarted belongingness significantly mediated the association between interpersonal negative life events and next-day suicidal thoughts.
Glenn 2022 [20]	53 adolescents that had recently received acute psychiatric care for suicide risk	14.85 (12–18)	64.2%	28 days of interval (2/day), signal (at least 3/day) and event (self-initiate when self-injurious thoughts and behaviours experienced) sampling with mobile phone		NSSI, and Suicidal Thoughts and Behaviours	The study’s feasibility was supported by reasonable enrollment rates and good adherence to EMA surveys and actigraphy. Adolescents reported a positive overall experience, finding the questions understandable, and the surveys minimally burdensome. Suicide attempts and re-hospitalisations were unrelated to the study and occurred at rates comparable to those of other adolescents at the recruitment site. Clinicians found the study minimally burdensome and somewhat positive for themselves, their patients, and families.
Haliczer 2023 [72]	134 university students	21.01	100%	14 days of other sampling: completed as close to end of day as possible (1/day)	83.1%	Self-injury (2 items)	The NSSI (compared to the no NSSI) group showed significantly higher self-conscious and negative emotional reactions to daily social stressors, and social stressors characterised by greater dysfunction. In the NSSI group, experiencing social stressors characterised by greater distress (relative to one’s average) was associated with same-day NSSI urges and behaviour, greater confusion predicted same-day NSSI urges, and greater conflict predicted same-day NSSI behaviour. Greater self-conscious and negative emotional reactions to these stressors predicted same-day NSSI urges and behaviour.
Hamilton 2024 [29]	60 high school students	16.15 (14–17)	49%	8 weeks of signal (3/day) sampling with mobile phone	86% of all study days had at least 1 EMA prompt completed	Passive Death Wish (1 item [73]) and Active Suicidal Ideation (1 item [73])	The results highlighted a nuanced relationship where social media both increases and reduces the risk of suicidal ideation on a daily basis. Adolescents reported lower suicidal ideation on days they felt more supported or encouraged on social media, while more frequent negative social media experiences were associated with higher suicidal ideation.
Horowitz 2018 [74]	38 self-injuring community young people	21.9 (18–30)	89%	21 days of signal (1/day) sampling		NSSI Urges and Acts	Significant associations between trauma severity and the following functions of NSSI: affect regulation, interpersonal boundaries, self-care, anti-dissociation, and marking distress. Participants with higher trauma severity tended to endorse more NSSI functions.
Hughes 2019 [26]	47 self-injuring young people	19.1 (15–21)	68%	2 weeks of signal (5/day) and event (self-initiate after NSSI thought or behaviour) sampling with mobile phone		NSSI Thoughts and Behaviours (8 items)	Higher reported levels of anxiety, feeling overwhelmed, overall negative affect, and repetitive negative thinking predicted more NSSI behaviours reported at the next assessment. Greater NSSI thought intensity ratings at the prior assessment also predicted more NSSI behaviours reported at the following assessment. Anxiety and feeling overwhelmed predicted NSSI most strongly when repetitive negative thinking was elevated.
Janssens 2023 [75]	1210 school students	13.8 (12–19)	66%	6 days of signal (10/day) sampling with mobile phone		Self-Harm Thoughts and Behaviours (3 items)	Higher paternal and maternal attachment insecurities were significantly associated with current self-harm thoughts, while peer attachment insecurity was not.
Jiang 2023 [76]	106 young people with suicidal thoughts	20.93 (18–25)	81.1%	8 weeks of signal (4/day) sampling with mobile phone	62.1%	Suicidal Ideation (2 items based on [54])	The EMA and wearable sensor protocol were feasible and acceptable in this sample. Adherence decreased over time. Greater baseline levels of hopelessness were associated with higher EMA adherence. Previous-day suicidal ideation predicted lower wearable sensor adherence on the next-day.
Kiekens 2020 [77]	30 university students with frequent past-year NSSI	20.1	80%	12 days of signal (up to 8/day) sampling with mobile phone	median 79.2%	Momentary NSSI Thoughts and Occurrence of NSSI Behaviour (4 items)	Negative affect was positively associated with NSSI thoughts at the same assessment, whereas positive affect and self-efficacy to resist NSSI were each negatively associated with NSSI thoughts. Higher-than-usual negative affect and self-efficacy to resist NSSI were predictive of short-term change in NSSI thoughts. Self-efficacy to resist NSSI predicted a lower likelihood of NSSI behaviour, even when controlling for NSSI thoughts.
Kim 2023 [78]	60 adults with repetitive NSSI	22.47 (19–36)	93.3%	2 weeks of interval (4/day) sampling with mobile phone		NSSI, and Suicide Thoughts, Urges and Behaviours	The best-fitting model showed support for two NSSI subtypes: (1) substance abuse and suicide attempt, and (2) cutting and scratching. The substance abuse and suicide attempt subtype endorsed higher rates of lifetime suicide plans and attempts, more severe self-harm behaviour, borderline personality traits, anger, posttraumatic symptoms, and emotion regulation difficulties. They also experienced greater anger, rejection, loneliness, and helplessness in the EMA assessments, compared to the cutting and scratching subtype.
Kleiman 2017 [46]	54 adults with past-year suicide attempt	23.24 (18–44)	79.6%	28 days of signal (4/day) and event (self-initiate whenever participant has suicidal thoughts) sampling with mobile phone	62.8%	Suicidal Ideation (3 items)	Suicidal ideation varied dramatically over the course of most days: 94.1% of participants had at least 1 instance of intensity of suicidal ideation changing by a standard deviation or more from one response to the next. Risk factors for suicidal ideation, hopelessness, burdensomeness, and loneliness also varied considerably over just a few hours and correlated with suicidal ideation but were limited in predicting short-term change in suicidal ideation.
Kleiman 2018 [79]	51 adults with past-year suicide attempt	23.59 (18–38)	79%	28 days of signal (4/day) sampling with mobile phone	55%	Suicidal Thoughts (3 items)	Five distinct phenotypes of suicidal thinking emerged that differed primarily in the intensity and variability of suicidal thoughts. Participants whose profile was characterised by more severe, persistent suicidal thoughts were most likely to have made a recent suicide attempt.
Koenig 2021 [80]	37 female outpatients	15.48	100%	2 or 4 days of signal (12/day) sampling with mobile phone		Dysfunctional Behaviour (1 item) and Self-injury Urge (1 item)	Self-injury increased negative affect and reduced feelings of attachment (specifically to mothers) in the following hour. The urge to self-injure predicted self-injury in the next hour at the global and subject levels but not at the within-person level. Higher negative affect was linked to a greater likelihood of self-injury in the next hour across global, subject, and within-person levels.
Kranzler 2018 [81]	47 self-injuring young people	19.1 (15–21)	68.1%	2 weeks of signal (5/day) and event (self-initiate after engaging in NSSI behaviours) sampling with mobile phone	85.1% of participants completed at least 80% of signal-contingent entries	Self-injury Thoughts (3 items) and NSSI Behaviours (4 items)	Momentary changes in both negative and positive emotions predicted increased intensity of NSSI thoughts at the following assessment but only increases in negative emotion predicted NSSI behaviours. After NSSI behaviours, participants reported reduced negative emotions and increased positive emotions. The most commonly endorsed function of NSSI was “to stop or get rid of bad or negative feelings”.
Kuburi 2024 [82]	160 university students with past-year NSSI	19.75	83%	14 days of signal (1/day) sampling	median compliance (completing 12 or more entries) = 86%	NSSI Thoughts and Engagement (4 items)	Daily stressors significantly predicted same-day, but not next-day, NSSI thoughts and engagement. Difficulties with emotion regulation significantly moderated the effect of daily stressors on NSSI thoughts and engagement.
Kudinova 2024 [22]	158 adolescents after psychiatric hospitalisation	15.18 (13–18)	60.8%	3 weeks of signal (5/day) sampling with mobile phone		NSSI Behaviour (1 item)	Levels of anger at self and others were associated with a higher number of subsequent NSSI occurrences within a day, even after adjusting for participants’ age, sex assigned at birth, number of current psychiatric diagnoses, EMA response rates, and lifetime history of suicidal ideation. Increases in NSSI occurrences were linked to subsequent increases in feelings of anger at self and feelings of shame.
Kuehn 2021 [83]	60 young people who self-harm or with past-year suicide attempt	18.58	76.7%	14 days of signal (5/day) and event (self-initiate when experiencing a significant increase in self-injurious thoughts or behaviour) sampling with mobile phone	87.4%	Self-Injurious Thoughts and Behaviour (9 items based on [32])	Suicidal thoughts were correlated with sadness, shame, guilt, fear, rumination, suppression, self-invalidation, distraction, and momentary urgency. Both within- and between-person negative emotions were predictive of suicidal thoughts, but not self-harm thoughts.
Lavis 2022 [84]	Young people with eating disorder diagnosis			14 days of signal (6/day) sampling with mobile phone		Self-harm Thoughts and Behaviours (4 items based on [32])	N/A (protocol).
Lear 2019 [85]	47 university students with past-year self-injurious episode	19.87	91.5%	2 weeks of signal (1/day) sampling with mobile phone	91.8%	NSSI Urge and Behaviour (3 items based on [86])	Self-criticism did not directly predict self-injury outcomes, but did indirectly predict urge intensity through daily thoughts about punishment. Daily guilt predicted self-injury urge intensity beyond daily sadness, hostility and fear and was the only type of negative affect associated with self-injury behaviour.
Mereish 2023 [21]	92 minority youth (Sexual and gender minority participants)	16.45 (12–19)	cisgender female = 35%	28 days of signal (1/day) sampling with mobile phone or computer	76%	Suicidal Ideation and Behaviours (2 items based on [60]) and NSSI Ideation and Behaviours (3 items based on [60])	On days minority young people experienced external and internalised minority stressors (i.e., identity concealment and internalised stigma), they reported greater intensity of suicidal and nonsuicidal self-injurious ideation and affective distress. Increased negative affect, lower positive affect and increased emotion dysregulation were associated with higher suicidal and nonsuicidal self-injurious ideation intensity on the same day. Within-person associations between minority stressors and intensity of suicidal ideation and NSSI ideation were mostly accounted for by negative affect and emotion dysregulation.
Miller 2019 [87]	40 young females with NSSI history	21.56 (18–25)	100%	14 days of interval (1/day) sampling with mobile phone	88%	NSSI Thoughts and Acts (2 items based on [60])	NSSI thoughts were positively associated with NSSI acts. Higher-than-usual daily perceived stress predicted same-day NSSI. Higher-than-usual stress did not predict next-day NSSI risk after accounting for same-day NSSI and next-day stress.
Molaie 2022 [88]	197 university students	19.4 (18–25)	79.2%	2 weeks of signal (1/day) sampling with mobile phone	81.9%	Suicidal Ideation (1 item based on [89])	Emotional satisfaction in daily interactions and the number of conflicts both predicted thwarted belongingness and suicidal ideation.
Nock 2009 [32]	30 young people with recent NSSI history	17.3 (12–19)	86.7%	14 days of signal (2/day) and event (self-initiate whenever experiencing self-destructive thoughts or behaviour) sampling with PDA device	83.3% completed at least 28 entries	Presence of Self-Injurious/Self-Destructive Thoughts (4 items), Presence of Self-Injurious/Self-Destructive Behaviour (5 items) and Functions of Self-Injurious/Self-Destructive Behaviour (14 items)	NSSI behaviour was associated with a shorter duration of NSSI thoughts. When NSSI thoughts were present, the occurrence of NSSI behaviour was predicted by greater thought intensity. During episodes of NSSI thoughts, being alone was a significant predictor of NSSI behaviour. The likelihood of engaging in NSSI were significantly increased when feeling rejected, anger toward oneself, self-hatred, numb/nothing, and anger towards another, but decreased in the presence of feeling sad/worthless.
Nugent 2022 [90]	194 adolescents after psychiatric hospitalisation	15.14 (13–18)	60.3%	3 weeks of signal (5/day) and event (self-initiate whenever participants experienced feeling strong suicidal ideation or had the urge to engage in some type of self-harm or suicidal behaviour) sampling with mobile phone		Suicidal Ideation and Behaviour, and Non-suicidal Self-Injury Thoughts and Behaviour	N/A (protocol).
Oakey-Frost 2023 [91]	49 university students	19.2	71.4%	10 days of signal (5/day) sampling with mobile phone	83.6%	Suicidal Desire (1 item), Wish to Live (1 item) and Wish to Die (1 item)	Wish To Die and suicidal ambivalence were associated with same-day and next-day suicidal desire. Wish To Live demonstrated a same-day protective relationship with suicidal desire and a positive next-day relationship with Wish To Die.
Portillo-Van Diest 2023 [92]	782 university students			15 days of signal (4/day) sampling with mobile phone	77%	Suicidal Ideation	N/A (protocol).
Ratzon 2024 [93]	29 psychiatric inpatients	14.79 (12–18)	100%	10 days of interval (2/day) sampling with pen and paper in clinical setting		Death Wish (1 item based on [94]), Suicidal Intent (1 item based on [94]) and Suicidal Intent Severity (1 item based on [94])	There was a significant positive association between sleep onset latency and expressing a “death wish” the following day, irrespective of sleep medication use and self-reported depression severity. Also a significant negative association between total sleep time and expressing a “death wish” the following day was observed.
Reeves 2022 [95]	10 adolescents with suicidal thoughts and in mental health treatment	(13–19)	40%	14 days of interval (9/day) sampling with mobile phone	82%	Suicidal Ideation (4 items based on [54])	The four suicidal ideation characteristics measured (desire to die, deterrents, controllability, and intent) varied significantly over the course of hours, days, and weeks. The suicidal ideation risk factors assessed (loneliness, feeling ignored, burdensomeness, feeling excluded, closeness, anger, and hopelessness) also showed significant variation. Desire to die was consistently related to rapid, subsequent decreases in the ability to control thoughts of suicide. Lastly, there were bi-directional relationships between suicidal ideation characteristics and suicidal ideation risk factors, suggesting that not only did risk factors influence suicidal thought but fluctuations in suicidal thought had an important impact on factors such as loneliness, anger, and hopelessness as well.
Schatten 2021 [96]	34 adolescents after psychiatric hospitalisation	15.5 (13–17)	76.5%	28 days of signal (1/day) sampling	69%	Suicidal Ideation (1 item)	Adolescents reporting higher misery and lower happiness relative to others had a significantly increased likelihood of same-day suicidal ideation. Individuals with elevated same-day, but not previous-day, misery and anger showed increased suicidal ideation. Elevated within-person happiness was protective for same-day suicidal ideation but was associated with higher next-day suicidal ideation.
Selby 2014 [97]	30 self-injuring young people	17.3 (12–19)	86.7%	14 days of signal (2/day) and event (self-initiate for self-destructive thought or behaviour) sampling with PDA device	83.3%	NSSI Thoughts (2 items), NSSI Behaviour (4 items) and Suicidal Thoughts (2 items)	Individuals with automatic positive reinforcement motivation reported higher NSSI thoughts, longer thought duration, and increased NSSI behaviours. They also reported more alcohol use thoughts, alcohol use behaviours, impulsive spending, and binge eating. In contrast, participants who reported trying to feel satisfaction during NSSI reported significantly less NSSI behaviour than those who did not. Individuals reporting actually feeling pain reported more NSSI behaviour than the other three motivations. Individuals who reported actually feeling stimulation, satisfaction, or some other sensation during NSSI did not report significantly different levels of NSSI than those who did not.
Selby 2021 [98]	47 actively self-injuring young people	19.1 (15–21)	68%	2 weeks of signal (5/day) and event (self-initiate entries after experiencing NSSI thought or behaviour) sampling with mobile phone		Dysregulated Behaviour (1 item; including NSSI)	Temporal Bayesian Network analysis showed strong support for the emotional cascade model, with high accuracy in predicting BPD diagnosis (around 90%) and reliably predicting rumination, negative emotions, and dysregulated behaviours (70% to 100% accuracy, depending on specificity). These findings reinforce the idea that BPD may arise from the interaction between emotional cascades and dysregulated behaviours.
Shingleton 2013 [99]	30 young people with NSSI history	17 (12–19)	87%	14 days of event (self-initiate after self-destructive thoughts or behaviours) sampling with PDA device		Suicidal Thoughts/Behaviour and NSSI Thoughts/Behaviour	Binge/purge thoughts mostly co-occurred with other self-destructive thoughts. Perceived criticism and feelings of rejection/hurt were associated more often with binge/purge thoughts than with NSSI thoughts.
Storkel 2021 [100]	51 people with repetitive NSSI	23.92 (18–45)	100%	14 days of signal (5/day, with 8 on first day) and event (self-initiate after NSSI act) sampling with mobile phone	92%	NSSI Urge (1 item) and NSSI Behaviour (8 items)	Salivary beta-endorphin levels immediately before an NSSI act were significantly lower than directly after NSSI. There was a positive association between severity of the self-inflicted injury and beta-endorphin levels, but no significant association between beta-endorphin levels and subjectively experienced pain.
Turner 2016 [101]	60 adults with recent repeated NSSI	23.25 (18–35)	85%	14 days of signal (1/day) sampling with online survey in email	87.5%	NSSI Urges (5 items [89]) and NSSI Acts (7 Items [32])	Daily conflict was associated with higher same-day NSSI urges and greater likelihood of NSSI acts. Perceived support increased after disclosed NSSI acts but not after undisclosed ones. This perceived support was then also associated with stronger NSSI urges and greater likelihood of NSSI acts on the following day.
Turner 2016 [102]	25 young people with NSSI and disordered eating episodes during diary period	23.12 (18–35)	92%	14 days of interval (1/day) sampling	90.9%	NSSI Behaviour (1 item)	Participants were more likely to act on NSSI thoughts after arguments or feelings of rejection. NSSI days featured more intense evening negative moods compared to fasting days and greater morning fatigue compared to binge eating/purging days.
Victor 2014 [103]	84 university students with recent NSSI and university students without NSSI history	23.3 (19–43)	71.4%	14 days of other sampling: completed as close to end of day as possible (1/day) with online or paper diary		NSSI Behaviour (12 items [104])	The largest group differences in emotion between those with and without NSSI, were dissatisfied with self, ashamed and sad. After controlling for BPD symptoms, the largest relationships with NSSI history were for disgust, cheerfulness, and joy.
Victor 2019 [105]	62 women with past-year suicide risk or aggressive behaviour	22 (18–24)	100%	21 days of signal (7/day) sampling with mobile phone	76.1%	Self-injurious Urges (1 item) and Suicide Urges (1 item)	Within-person increases in internalising negative affect predicted later self-injurious urges. Rejection and criticism also predicted subsequent self-injurious urges. The impact of rejection and criticism on later NSSI and suicide urges was mediated by internalising negative affect, while rejection maintained a direct effect on NSSI urges.
Victor 2021 [106]	161 young women with past-year suicide risk or aggressive behaviour	21.52 (18–24)	100%	3 weeks of signal (7/day) sampling		NSSI Urge (1 item) and Suicide Attempt Urge (1 item)	Higher mean negative affect and negative affect variability were associated with all types of self-injurious thoughts and behaviour assessed prospectively or concurrently.
Vine 2020 [107]	162 adolescents receiving psychiatric or behavioural treatment	12.03 (11–13)	46.9%	4 days of signal (2–3/day) sampling with mobile phone		Suicide Risk (2 items)	Suicide risk was related to daily dissociative experiences, independent of daily negative and positive affect and co-occurring borderline personality symptoms. This was only significant in adolescent girls when examining demographic effects in the model.
Williams 2022 [108]	16 LGBTQ+ young people with self-harm thoughts or behaviour	19.2 (16–25)	62.5%	7 days of signal (6/day) sampling with mobile phone	67.6%	Self-harm Thoughts (1 item [109]), Self-harm Behaviour (1 item [109]) and Suicidal Thoughts (1 item [109])	The EMA design was feasible and acceptable among the sample. The pilot data demonstrated fluctuations of depression and anxiety symptoms.
Williams 2023 [110]	40 minority young people (21 LGBTQ+ young people in sample 1 and 19 black and ethnic minority young people in sample 2)	Sample 1 = 21.4 (16–25) Sample 2 = 21.1 (17–25)	Sample 1 = 67% Sample 2 = 84.2%	21 days of signal (1/day) sampling with mobile phone	Sample 1 = 80.7% Sample 2 = 78.9%	Self-harm Thoughts (1 item [109])	N/A (no EMA-related findings reported; results focused on intervention).
Wolford-Clevenger 2019 [111]	206 university students	19.05	70.6%	90 days of signal (1/day) sampling		NSSI, Suicidal Thoughts and Behaviour, and Perceived Capability for Suicide	Individuals with higher levels of suicidal ideation and hopelessness were more likely to drop out and not complete the final assessment. Participants who completed the final assessment had neutral to positive experiences in the study.

Note. BPD: borderline personality disorder, EMA: ecological momentary assessment, HC: healthy control, IL-6: Interleukin-6, NSSI: non-suicidal self-injury, PTSD: post-traumatic stress disorder, STD: student group.

**Table 2:** Quality Assessment Summary.

Reporting Quality Criteria	Study Compliance (*n*)			
	Full	Partial	No	N/A
EMA/ESM/AA/IL in title and keywords	27	22	30	
Rationale for method in introduction	60	7	12	
***Methods***				
Training for participants	17	21	41	
Compliance and incentives	59	8	12	
Technology specified	51	6	22	
Sample size justified	8	5	66	
Sampling design rationale	5	1	73	
Sampling density/frequency	8	15	56	
Technical details of sampling	2	6	71	
Design feature	6	3	70	
Full text of items	41	27	11	
Item reliability and validity	7	35	37	
Inclusion/exclusion criteria for data	6	18	52	3
Description of data cleaning and preparation	14	22	40	3
Statistical analysis	48	23	6	2
***Results***				
Description of data	38	22	15	4
Prompt delivery	1	4	69	5
Latency	1	2	71	5
Compliance rate	47	11	16	5
Missing data	9	8	58	4
***Discussion***				
Limitations	33	9	36	1
***Transparency and reproducibility***				
Pre- or post-registration	5	2	72	
Open materials	3	12	64	
Open code	3	4	68	4
Open data	2	19	56	2

Note. N/A = not applicable (e.g., studies that were limited to event-contingent sampling, published protocols).

### 3.3. Feasibility of EMA

The EMA studies showed high feasibility and acceptability across the adolescent samples, even in high-risk populations. Studies indicate that adolescents generally adhered well to the EMA protocols, found surveys to be understandable and minimally burdensome, and reported neutral to positive experiences [20,49,108,111]. While adherence often declined over time, EMA completion rates remained reasonable [20,30,47,51,76,83] and engagement did not appear to be adversely affected by response-contingent interventions [38]. However, researchers acknowledged that some adolescents may underreport severe suicidal ideation in EMA compared to interviews. Qualitative findings indicated that this underreporting reflected adolescents’ avoidance of further follow-up questions and concerns about potential consequences, such as parental notification or rehospitalisation [19]. Nevertheless, most adolescents perceived daily EMA diaries as acceptable, with many reporting no negative impact or even slight improvements in mood [30,76].

Further, EMA has been integrated into intervention studies, demonstrating initial feasibility in monitoring treatment outcomes and engagement. Studies have used EMA reporting of self-harm thoughts to identify the impact of emotion-focused and cognitive processing interventions [37,66]. Additionally, EMA has been used to assess engagement and adherence within randomised control trials [55,110]. In a trial investigating the feasibility and acceptability of Purrble, a socially assistive robot, EMA was employed to measure self-harm thoughts and engagement with the Purrble device each day [110]. Similarly, EMA was used to assess adolescents’ daily adherence to safety plans following hospital discharge in the MI-Safe-Cope trial, alongside measuring fluctuations in suicidal ideation and self-efficacy [55]. Collectively, these studies demonstrate the utility of EMA as an approach for assessing real-time engagement during the intervention period.

### 3.4. Compliance

The reported compliance rates across the studies vary greatly, ranging from 38% [36] up to 92% [85,100], with many studies achieving compliance in the 70–80% range. One study also reported a 99.9% response rate specifically in the healthy control group [41]. Table 1 includes compliance rates, or other indicators of data completion, where reported, for each study. There was no clear trend of the study duration or frequency of prompts impacting the compliance rate of EMA assessment completion. A variety of other factors play a role, including incentive structures, participant populations and logistical barriers. The EMA studies in this review implemented various incentive structures and strategies to maximise compliance and retention. Monetary compensation was a common approach, with 66 studies (83.5%) reporting that financial incentives were offered for study involvement. This was often tiered or conditional on achieving compliance thresholds (e.g., a bonus fee for an 80% compliance rate). Non-monetary rewards, such as course credits or retaining study devices, were also employed, e.g., [25,40,110].

Reminder systems, including automated repeat notifications for missed prompts, follow-up emails, and personal contact from researchers, were frequently used to encourage timely responses. A small number of studies also extended the data collection period when assessments were missed to ensure a minimum number of daily entries, e.g., [34,67]. Some studies in this review showed consideration to tailor the timing of EMA prompts to participants’ daily routines to maximise compliance (*n* = 21; 26.6%). These included aligning assessments with participants’ typical wake and bedtimes, avoiding school hours, and customising schedules based on individual availability and preferences. Customising EMA schedules to align with participants’ daily routines minimised intrusion into participants’ lives but also made data collection more feasible. For example, Reeves et al. [95] reported that their adolescent sample had an overall compliance rate of 82%; however, when accounting for assessments missed due to participants being asleep, this increased to 90% compliant. Further, studies that included follow-up questionnaires or interviews identified scheduling as a barrier to completing EMA assessments, e.g., [20,76]. Participants reported difficulties fitting prompts into their daily lives at times, especially between being busy at school or work and sleep [76].

### 3.5. Safety Planning and Monitoring

Given the nature of collecting real-time data on self-harm, researchers implemented safety monitoring protocols to protect participants’ well-being during data collection. However, over half of the reviewed EMA studies did not report detailed information about their safety planning and monitoring procedures. Responses to EMA surveys were often reviewed at regular intervals, daily or multiple times per day, to identify high-risk responses [21,30,32,70,95,110]. Some studies implemented automated systems that flagged high-risk responses, such as elevated suicidal intent or reported self-harm behaviour, to trigger alerts to clinicians or research team members [34,38]. These alerts or regular monitoring prompted follow-up actions, including risk assessments conducted via phone by trained clinicians or research assistants. Studies also incorporated further immediate safety planning, such as pop-up messages on high-risk responses within the EMA applications that directed participants to review pre-established safety plans or contact support resources, e.g., [38,77,88,108].

Many studies employed a tiered approach, where risk levels (e.g., low, moderate, high) dictated intervention intensity, ranging from providing additional resources to direct emergency services involvement. Collaboration with caregivers, outpatient clinicians, and guardians was reported, particularly for high-risk participants. Participants were often provided with crisis resources, including contact details for emergency services and helplines, before starting the EMA protocol or at the end of each assessment, e.g., [23,29,42,74,82]. Further, two records [69,91] outlined the use of the Virtual Hope Box mobile app as an additional resource to support emotion regulation during the study’s data collection period.

### 3.6. Predictors of Variability in Self-Harm

Suicidal ideation fluctuates dramatically across hours, days and weeks [46,95]. Associated risk factors, such as burdensomeness, hopelessness and loneliness, also differed significantly over time [42,95]. Increased NSSI thoughts predicted NSSI behaviour [24,26,32,87]. Similarly, the urge to self-injure predicted engaging in self-injury [80].

The form, functions, and motives of NSSI were highly variable, both between individuals and within the same person over time. Affect regulation emerged as a highly endorsed function in a number of studies, with participants reporting using self-harm behaviours to reduce negative emotion or distress [25,35,59,81,83,99,100]. However, the functions of NSSI were not static; individuals often exhibited shifts in the reasons they engage in self-injury, and some report multiple functions co-occurring within the same episode [25]. Further, participants with higher trauma severity tended to endorse more NSSI functions [74]. Those who engaged in NSSI for automatic positive reinforcement, such as seeking stimulation or relief, tended to have higher levels of self-injurious thoughts, longer thought duration, and greater engagement in other impulsive behaviours such as alcohol use, binge eating, and impulsive spending [97]. The most frequently endorsed method of self-harm across the studies was cutting, often followed by scratching or picking at wounds [24,26,35,36,37,39,81,83,85,100].

Momentary fluctuations in affective states were identified across studies to impact self-harm risk. Negative affect was consistently linked to self-harm as a significant predictor [26,40,43,81,87,96,106]. Individuals who engaged in NSSI typically experienced rising negative emotions before an episode, which peaked during the act and declined afterwards [34,36,81]. However, Koenig et al. [80] report an increase in negative affect in the hour following self-injury. EMA studies identified that self-harm was correlated with factors such as anxiety, feeling overwhelmed, self-hatred, repetitive negative thinking, anger, sadness, shame, guilt, frustration and fear [22,26,32,40,41,83,85]. Self-punishment and self-criticism were also identified as significant predictors of NSSI urges [42,85].

Burdensomeness was identified as a significant predictor of next-day suicidal ideation [27] and same-day ideation frequency and duration [56]. Additionally, feelings of thwarted belongingness within the family and among friends were directly associated with suicidal thoughts [71]. Hopelessness about one’s thwarted belongingness, interpersonal hopelessness about perceived burdensomeness on others and the interaction between these constructs were significant predictors of same-day and next-day suicidal desire [69].

Social support was related to NSSI urges, but not NSSI behaviours [23] and suicidal ideation [51]. Engaging in NSSI appeared to elicit increased perceived support when disclosed, yet this support was paradoxically associated with stronger urges and a higher likelihood of NSSI acts the following day [101]. The quality of social interactions also made an impact, with emotional satisfaction in daily interactions and the number of conflicts predicting suicidal ideation [88]. Daily conflicts further heightened self-harm risk, with arguments and rejection increasing the likelihood of acting on NSSI thoughts [102]. Being alone during NSSI thoughts was a strong predictor of NSSI acts, and the likelihood of NSSI increased with feelings of rejection, self-hatred, and anger towards others [32]. Feelings of exclusion predicted same-day increases in suicidal ideation [65]. Similarly, rejection and criticism significantly increased the likelihood of later self-injurious urges [105].

Seeking professional support and finding coping strategies helpful were associated with lower next-day suicidal urges, though professional support was the least utilised strategy [31]. Adolescents frequently used NSSI to cope with suicidal thoughts, but those who employed a greater number of coping strategies and perceived them as helpful were less likely to engage in NSSI [18].

Stress strongly predicted same-day NSSI thoughts and behaviour, with emotion regulation difficulties amplifying this effect [72,82,87]. Furthermore, young adults with a recent history of NSSI exhibit heightened negative emotions to daily stressors [39].

Individuals with a history of repetitive NSSI were more likely to experience irregular sleep patterns, which in turn predicted greater daily urges to self-harm, even after accounting for other sleep-related factors and negative affect [45]. Similarly, difficulties falling asleep, nightmares, and pre-sleep rumination were associated with heightened suicidal thinking the following day [70]. Prolonged sleep onset latency was also linked to an increased likelihood of expressing a “death wish” the next day, whereas longer total sleep time appeared to have a protective effect [93].

## 4. Discussion

This review identified 79 papers that used EMA methods to explore adolescent self-harm. The included articles highlight an ongoing increase in published adolescent self-harm EMA studies since 2009, with significant growth observed after 2018. This upward trend is expected to continue, as supported by ongoing and unpublished studies identified during the search. Rodriguez-Blanco et al. [13] review concluded that EMA is a valuable yet underutilised tool in this field, with a particular lack of research in adolescent samples. However, the findings of this current review demonstrate a substantial increase in recognising and leveraging EMA within this field of research. The increasingly widespread availability of smartphones supports the ability to record data in real time, with accurate time-stamped entries, and to collect data in adolescents’ everyday environments [13,14]. This growing body of work provides greater insight into the nuances and short-term nature of self-harm risk and protective factors.

The majority of the research evidence base in this review assessed correlates and predictors of self-harm with the EMA data. Specifically, this review highlights that self-harm urges and behaviours exhibit substantial within-person variability [46,95]. Adolescents engaging in self-harm thoughts and behaviour often experience rapid shifts in mood, suggesting that traditional retrospective methods may not be capturing the fluctuations occurring in short-term periods. Negative affect is frequently assessed in the literature and shows predictive value for self-harm risk, e.g., [26,36,96]. Other significant predictors included burdensomeness, thwarted belongingness, self-punishment, guilt, stress, irregular sleep, anxiety and frustration.

### 4.1. Limitations and Challenges of Conducting EMA

The articles reviewed demonstrate that EMA is a feasible and acceptable research method for adolescents, though compliance rates vary considerably, and participant burden remains a concern [20,47]. To mitigate fatigue from repeated assessments, several studies incorporated daily diary designs as a less intrusive alternative. Although EMA was also generally well-tolerated, safety planning and monitoring protocols were inconsistently reported across studies. Some studies used automated risk alerts and follow-up calls, while others relied on self-guided safety plans. The diverse approaches to safety and monitoring attempted to balance participant autonomy, confidentiality, and ethical safety monitoring, highlighting the need for tailored strategies that reflect study contexts and participant vulnerabilities. However, a majority of the studies did not provide clear details on their risk management procedures, preventing a comprehensive evaluation of their effectiveness in ensuring participant safety. This lack of transparent reporting limits the ability to compare protocols across studies and identify best practices for managing risk in real-time assessments.

Additional considerations for adolescent samples included avoiding prompts during prohibited smartphone use times, such as school hours, and tailoring survey windows to fit within their normal waking hours or activities. In some studies, intervals for completing surveys were considered to ensure ample completion time while avoiding interference with participants’ schedules. Overall, these personalised approaches highlight the importance of adapting EMA protocols to enhance feasibility, data quality, and participant compliance, particularly in contexts with specific constraints such as school settings.

### 4.2. Limitations of the Current Review

There is significant heterogeneity in EMA protocols across the included studies, with variations in sampling strategies (e.g., event-contingent vs. time-based assessments), frequency of prompts and total duration of monitoring making direct comparisons difficult. This methodological diversity can complicate cross-study comparisons and prevent the consolidation of findings, thereby limiting the potential for robust meta-analyses or systematic evaluations. Additionally, the choice of measures in EMA research often reflects a compromise between comprehensive assessment and participant burden. While brief or single-item measures can be advantageous for minimising fatigue and burden, the absence of validated instruments raises questions about the reliability and interpretability of study outcomes. Greater methodological consistency and transparent reporting are needed to ensure comparability and replication across studies. A further limitation is the considerable variability in the terminology used to capture self-harm in this area (including NSSI, self-harm, suicidal thinking). This creates challenges in comparing findings across studies that define or operationalise these constructs differently.

The concentration of studies in high-income Western countries, particularly the United States, underscores a significant gap in EMA research on self-harm behaviours within low- and middle-income countries. This uneven concentration of research samples limits the generalisability of findings, as cultural and socioeconomic differences may result in distinct self-harm risk factors [5,112,113]. Moreover, the feasibility of conducting EMA in resource-limited settings may be more challenging with these factors impacting recruitment and retention, as well as the broader applicability of EMA-derived insights.

Finally, only one reviewer conducted the quality assessment for each study included in this review. Despite uncertainties being resolved through consulting with a second reviewer, this may introduce rater bias for the quality assessment.

### 4.3. Future Research Directions

Future research on adolescent self-harm using EMA would benefit from establishing more rigorous and transparent safety protocols. Although early detection of risk factors presents an important advantage of real-time monitoring, clear guidelines for appropriate intervention within the research setting are lacking [114,115]. Researchers should detail the processes by which elevated risk is identified (e.g., thresholds for alerting clinical teams or guardians) and specify the steps taken to safeguard participants, especially when adolescents report self-harm behaviour.

Along with these considerations for clear risk monitoring practices, the field would benefit from developing and validating standardised EMA measures that capture self-harm thoughts, behaviours, and relevant psychological factors. Methodological heterogeneity remains a major obstacle; inconsistencies in measure selection, sampling frequency, and analytical approaches have fragmented the literature, preventing the quantitative synthesis of results in this review. Validated scales could improve data quality, while also facilitating future meta-analyses abilities and enable pooling of data sets [17].

Despite the breadth of constructs assessed across studies, several notable gaps remain. First, self-harm is inconsistently and often narrowly operationalised. Most studies focus on either suicidal ideation or NSSI in isolation, rarely assessing both together, e.g., [20,21,90] or adopting a broader definition of self-harm, such as in the NICE [2] guidelines [33,75,84,110]. Secondly, sleep remains an underexplored proximal predictor. Although cross-sectional studies have linked disturbed sleep and insomnia to self-harm risk [116], only a small number of EMA studies have explored sleep variables. Given the developmental significance of sleep changes in adolescence [117,118] and preliminary EMA evidence linking poor sleep to next-day increases in suicidal ideation [70,93], this is a key area for future intensive longitudinal research. Third, the assessment of protective factors is sparse; most EMA studies to date have prioritised risk factors, with little attention on fluctuations in constructs like connectedness, coping strategies and help-seeking. Incorporating repeated measures of such protective factors, alongside risk, would provide a more balanced account and may identify real-time opportunities for intervention.

Another important avenue for future research is the effectiveness of EMA-based interventions [119,120]. While EMA is primarily utilised as an observational study method, it also has the potential to serve as a platform for just-in-time adaptive interventions (JITAIs). By capturing real-time fluctuations in self-harm risk, EMA could facilitate the timely delivery of targeted support [53]. Current evidence indicates that adolescents typically find smartphone-based EMA approaches acceptable, suggesting opportunities for its integration into both clinical and community settings. Leveraging this, clinicians may incorporate brief, real-time self-monitoring tools within existing therapeutic frameworks, allowing for continuous risk assessment, dynamic intervention adjustments, and potentially reducing future self-harm behaviours.

## 5. Conclusions

There is a growing interest in the capacity of EMA methods to reduce recall bias, increase ecological validity and provide detailed insights into the short-term risk and protective factors of adolescent self-harm in recent years. Furthermore, the digital habits and smartphone use among adolescents underscore EMA feasibility, provided that researchers employ rigorous safety monitoring protocols and minimise participant burden. Nonetheless, current studies show considerable methodological heterogeneity, including variability in sampling schedules and measures, thus limiting cross-study comparisons. Future investigations need to focus on standardising EMA protocols, diversifying samples within the field and continuing to address the challenges of conducting real-time research. By improving our ability to capture fluctuations in risk, this research method may support more timely and targeted interventions, significantly refining suicide prevention strategies.

## Data Availability

No new data were created or analysed in this study. Data sharing is not applicable to this article.

## Supplementary Materials

The following supporting information can be downloaded at https://www.mdpi.com/article/10.3390/ijerph23010084/s1, Supplementary File S1: PRISMA 2020 Checklist [121].

## Appendix A

Search terms

(“ambulatory assessment*” OR “experience sampl*” OR “ecological momentary assessment” OR “EMA” OR “ESM” OR “intensive time sample” OR “momentary time sampling” OR “real-time assessment” OR “daily diary” OR “daily task*” OR “daily activit*” OR “context-aware experience*” OR “event-contingent” OR “signal-contingent” OR “self-monitor” OR “self-regulat*” OR “self-aware”)

AND

(“suicide*” OR “self?injur*” OR “NSSI” OR “nonsuicidal self directed violence” OR “parasuicide” OR “self?destruct*” OR “self?harm” OR “selfharm” OR “DSH” OR “self?mutilat*” OR “automutilat*” OR “self?inflict*” OR “self?immolat*” OR “self?poison*” OR “overdose*” OR “cutt*” OR “self-cutting”)

AND

(“young adult*” OR “youth” OR “college student*” OR “university student*” OR “emerging adult*” OR “adolescence” OR “adolescent” OR “high school students” or “secondary school students” OR “post secondary students” OR “teen*” OR “juv*”)

*Adapted from* [12]
